# Phosphoproteomic Analysis of *Haemaphysalis longicornis* Saliva Reveals the Influential Contributions of Phosphoproteins to Blood-Feeding Success

**DOI:** 10.3389/fcimb.2021.769026

**Published:** 2022-01-18

**Authors:** Desmond O. Agwunobi, Ningmei Wang, Lei Huang, Yefei Zhang, Guomin Chang, Kuang Wang, Mengxue Li, Hui Wang, Jingze Liu

**Affiliations:** ^1^ Ministry of Education Key Laboratory of Molecular and Cellular Biology, Hebei Key Laboratory of Animal Physiology, Biochemistry and Molecular Biology, College of Life Sciences, Hebei Normal University, Shijiazhuang, China; ^2^ Hebei Xiaowutai Mountain National Nature Reserve Management Center, Zhangjiakou, China

**Keywords:** blood-sucking arthropods, host immune modulation, microscopy, RNA interference, tick salivary phosphoproteome, tick bite site, vectors

## Abstract

Tick saliva, an essential chemical secretion of the tick salivary gland, is indispensable for tick survival owing to the physiological influence it exerts on the host defence mechanisms *via* the instrumentality of its cocktail of pharmacologically active molecules (proteins and peptides). Much research about tick salivary proteome has been performed, but how most of the individual salivary proteins are utilized by ticks to facilitate blood acquisition and pathogen transmission is not yet fully understood. In addition, the phosphorylation of some proteins plays a decisive role in their function. However, due to the low phosphorylation level of protein, especially for a small amount of protein, it is more difficult to study phosphorylation. Maybe, for this reason, the scarcity of works on the phosphorylated tick salivary proteomes still abound. Here, we performed a phosphoproteomic analysis of *Haemaphysalis longicornis* tick saliva *via* TiO_2_ enrichment and the most advanced Thermo Fisher Orbitrap Exploris 480 mass spectrometer for identification. A total of 262 phosphorylated tick saliva proteins were identified and were subjected to functional annotation/enrichment analysis. Cellular and metabolic process terms accounted for the largest proportion of the saliva proteins, with the participation of these proteins in vital intracellular and extracellular transport-oriented processes such as vesicle-mediated transport, exocytic process, cell adhesion, and movement of cell/subcellular component. “Endocytosis”, “Protein processing in endoplasmic reticulum”, and “Purine metabolism” were the most significantly enriched pathways. The knockdown (RNAi) of Tudor domain-containing protein (TCP), actin-depolymerizing factors (ADF), programmed cell death protein (PD), and serine/threonine-protein kinase (SPK) resulted in the dissociation of collagen fibers and the pilosebaceous unit, increased inflammatory infiltrates/granulocytes (possibly heterophiles), and the depletion of the epithelium. Ticks injected with SPK dsRNA engorged normally but with a change in skin colour (possibly an autoimmune reaction) and the failure to produce eggs pointing to a possible role of SPK in reproduction and host immune modulation. Ticks injected with ADF dsRNA failed to acquire blood, underscoring the role of ADF in facilitating tick feeding. The results of this study showed the presence of phosphorylation in tick saliva and highlight the roles of salivary phosphoproteins in facilitating tick feeding.

## Introduction

Ticks are obligate blood-sucking arachnids that utilize their salivary constituents to physiologically influence the defence mechanisms of the host (Cabezas-Cruz and Valdés, 2014). Ticks, being the most important vectors of pathogens affecting animals, are of great veterinary importance, and also, constitute enormous public health importance, having been considered only second to mosquitoes as the most significant vectors of human diseases (Dantas-Torres et al., 2012). Their capacity to obtain large volumes of blood over a relatively long period, their longevity and high reproductive capacity, as well as having a broad host spectrum for many species substantially contribute to their success as disease vectors (Šimo et al., 2017).

To obtain a blood meal, ticks target a variety of vertebrates including mammals, birds, reptiles, and amphibians by piercing the skin and inserting their specialized mouthparts, then hold on tightly while they draw blood for days depending on the species (Sonenshine, 1992). Ticks are pool feeders in that they inflict injury on the capillaries and small blood vessels with their specialized mouthparts to create a haemorrhagic pool from which they ingest blood (Kazimírová and Štibrániová, 2013). Tick feeding activity involves the injection of saliva and the absorption of blood meal in an alternating manner *via* the same canal (Šimo et al., 2017). While feeding, the ticks pick up pathogens in the infected host. When the pathogens get to the midgut, they spread to the haemocoel by escaping through the digestive epithelium and then extending to the salivary gland by crossing the salivary gland epithelium. Thus, as the saliva is being injected during tick feeding, tick-borne pathogens can be transmitted to a new host (Nuttall and Labuda, 2008; Šimo et al., 2017). This mode of pathogen transmission promoted *via* arthropod/tick saliva during blood acquisition has been described as saliva-assisted transmission (SAT) (Nuttall and Labuda, 2008).

Tick saliva is comprised of a wide range of physiologically and pharmacologically active ingredients (proteins, peptides, molecules, etc.) that are indispensable for attachment to host, obtention of blood meal, and pathogen transmission, among others (Ramamoorthi et al., 2005; Hovius et al., 2008). These salivary constituents interact with the host processes such as immunity and inflammation, coagulation and fibrinolysis, angiogenesis, etc. (Brossard and Wikel, 2004; Fukumoto et al., 2006; Maritz-Olivier et al., 2007). The complexity and functional redundancy observed in the composition of the tick saliva are characteristics that developed during the host-parasite co-evolution to counter the complexity and redundancy of the host defence responses (Brossard and Wikel, 2008; Francischetti et al., 2009; Fontaine et al., 2011).

Previous studies have identified several tick saliva proteins of different categories including protease inhibitors such as alpha-2-macroglobulin, serpin, and cystatin; proteases such as metalloprotease, cathepsin, and trypsin-like proteases; heme metabolism-associated proteins such as ferritin, hemelipoprotein; heat shock proteins (protein modification machinery); proteins associated with metabolic processes, transporter activity, and structural functions, among others (Díaz-Martín et al., 2013; Oliveira et al., 2013; Mudenda et al., 2014; Tirloni et al., 2014). In fully engorged ticks, some proteins that are described as anticoagulant molecules including haemalin (Liao et al., 2009), microphilin (Ciprandi et al., 2006), and BmAP (Horn et al., 2000) have been identified. A study showed that the binding of tick saliva protein sialostatin L2 to annexin A2 inhibits the formation of NLRC4 inflammasome during infection with *Anaplasma phagocytophilum* (Wang et al., 2016). In addition, mounting evidence has shown the presence of various extracellular vesicles (EVs)/exosomes in tick saliva, which are believed to mediate pathogen transmission (Hackenberg and Kotsyfakis, 2018). EVs have been implicated to mediate the transmission of tick-borne Langat virus (LGVT) as tick cell-derived exosomes were found to contain LGVT RNA and Nonstructural 1 (NS1) protein (Zhou et al., 2018). Vital proteins which include thioredoxin peroxidase, heat shock proteins, proteases, and glyceraldehyde 3-phosphate dehydrogenase (GAPDH) have been identified in tick-derived EVs as revealed *via* proteomic analysis (Nawaz et al., 2020). It was hypothesized that tick salivary exosomes convey tick microRNAs and proteins to their host to influence definite biological functions (Hackenberg and Kotsyfakis, 2018). This hypothesis was confirmed by the identification of synaptobrevin (a crucial vesicle- and exocytosis-associated protein collectively called v-SNARES) in *Amblyomma americanum* and the observation of low tick engorgement weights, increased tick mortality, and premature tick detachment from the host in synaptobrevin-silenced ticks underscore their vital contribution to tick feeding success (Karim et al., 2005). A recent study demonstrated that EVs from *Ixodes scapularis* enhance tick feeding and facilitate infection of *Anaplasma phagocytophilum via* SNARE proteins (Vamp33 and synaptobrevin 2) and dendritic epidermal T cells, whereas, *Dermacentor andersoni* EVs mitigate microbial spreading of the pathogen *Francisella tularensis* (Oliva Chávez et al., 2021).

The hard tick *Haemaphysalis longicornis* is a vector of medical and veterinary importance and it is widely distributed in Eastern Asian countries including China, Japan, Korea, Australia, and New Zealand (Hoogstraal et al., 1968; Tirloni et al., 2015). The presence of *H. longicornis* was first reported in New Jersey in the United States of America in 2017, but has recently been reported in 11 more states (Rainey et al., 2018; Duncan et al., 2020; USDA, 2020). *H. longicornis* has been implicated as a vector of several diseases, transmitting pathogens such as the severe fever with thrombocytopenia syndrome virus (SFTSV) (Luo et al., 2015), *Rickettsia* spp. (Uchida et al., 1995; Tabara et al., 2011), *Ehrlichia chaffeensis* (Sun et al., 2008), *Coxiella burnetii* (Lee et al., 2004), *Anaplasma bovis* (Lee and Chae, 2010), and other pathogens of veterinary importance namely *Theileria* spp. (Li et al., 2009), and *Babesia* spp. (Guan et al., 2010), the causative agents of theileriosis and babesiosis, respectively.

Although the saliva proteome of *H. longicornis* has been previously analysed and catalogued (Tirloni et al., 2015), many of the proteins are still unidentified and their functions remain undefined. In addition, to the best of our knowledge, no study has been carried out on the phosphorylated saliva proteome. Given that post-translational modifications increase the functional diversity of proteome, the present study identified and analysed vital phosphorylated saliva proteomes of *H. longicornis* ticks to provide insight on their functions, particularly on how they facilitate tick feeding and pathogen transmission. Four proteins were selected for RNA interference experiment given the vital roles they play in other organisms/arthropods as reported in the literature. In this study, the most advanced Thermo Fisher Orbitrap Exploris 480 mass spectrometer was used to identify these phosphorylated saliva proteins in order to find more functional phosphorylated proteins in tick saliva.

## Materials and Methods

### Tick Feeding and Saliva Collection

The *H. longicornis* ticks used in the present study were collected *via* the flag dragging method from the vegetation of Xiaowutai Mountain National Nature Reserve in Hebei Province, China. These ticks were raised in an artificial climate incubator at 25 ± 1°C and 75% relative humidity (16/8 h of light/dark cycle) when they were not sucking blood. Then, they were moved into the cloth earmuffs over the New Zealand white rabbit’s ear for sucking of blood. When the female ticks became semi-engorged (96 h after attachment) from blood sucking, they were pulled from the rabbit’s ears. The semi-engorged stage was chosen given that it is the fastest feeding period with the secretion of a large number of proteins assisting blood sucking in the saliva, and it takes 1-2 days from this feeding stage to full engorgement. Then the ticks’ mouthparts were wiped with 75% alcohol and dopamine were injected into their bodies using a Hamilton syringe (33-gauge needle) within a short time. Shortly, the ticks began to secrete saliva, which was collected by capillary tube and quickly transferred into the centrifuge tube with sterile PBS (1 M, pH=6.8) containing protease inhibitor cocktail (Roche Ltd. Basel, Switzerland, 1 tablet/50 ml PBS) and phosphatase inhibitor cocktail (Thermo Fisher Scientific, USA 1 tablet/50 ml PBS), and then quickly frozen in liquid nitrogen and kept in –80°C. About 800 female ticks were used, and about 850 μl of saliva was extracted in this experiment. All experimental procedures were approved by the Animal Ethics Committee of Hebei Normal University (Protocol Number: 165031).

### Sample Preparation

After the frozen saliva was melted, firstly, reductive alkylation was performed for the identification of phosphorylated proteins. The samples were sonicated on ice for 2 min and then kept for 30 min on ice. Centrifugation was performed at 15,000 rpm for 15 min at 4°C, after which the supernatant was collected and transferred to a new tube, and the final volume was adjusted to 1 ml with 8 M urea. 20 µl of 0.5 M TCEP was added and the sample was incubated at 37°C for 1 h, and then 40 µl of 1 M iodoacetamide was added to the sample. Then, it was incubated at room temperature for 40 minutes while protected from light. To precipitate the proteins overnight at –20°C, five volumes of –20°C pre-chilled acetone were added. After that, the precipitates were washed twice by 1 ml pre-chilled 90% aqueous acetone solution, and then redissolved in 1 mL 100 mM TEAB. The proteins were digested at 37°C overnight after the addition of sequence grade modified trypsin (Promega, Madison, WI) at the ratio of 1:50 (W: W). The peptide mixture was desalted by C18 ZipTip and then lyophilized. The phosphopeptides were selectively enriched with High-Select™ TiO_2_ Phosphopeptide Enrichment Kit (Thermo Fisher Scientific, USA) according to the manufacturer’s guidelines. The enriched phosphopeptides were lyophilized.

### Nano-UHPLC-MS/MS Analysis

The peptides were redissolved in solvent A (A: 0.1% formic acid in water) and analysed by LC-MS which consists of EASY-nano LC 1200 system (Thermo Fisher Scientific, USA) and Orbitrap Exploris 480 (Thermo Fisher Scientific, USA). 3 µL sample was loaded into analytical column (Acclaim PepMap C18, 75 μm × 25 cm, Dionex, USA) and separated with 130 min-gradient. A flow rate of 250 nL/min was maintained using a linear ACN gradient of 2~8% solvent B in 6 min followed by 8% to 35% solvent B in the next 124 min (solvent A: 99.9% H_2_O, 0.1% formic acid; solvent B: 99.9% ACN, 0.1% formic acid). The mass spectrometer was operated under the data-dependent acquisition mode. The parameters were set as follows: (i) The electrospray voltage was set 2 kV; (ii) MS: scan range (m/z) = 350-1200; resolution=120,000; AGC target=300; maximum injection time=50 ms; (iii) HCD-MS/MS: resolution=30,000; AGC target=200; collision energy=35.

### Data Analysis

Raw Data was processed and analysed by Spectronaut 14 (Biognosys AG, Switzerland) with default settings. Spectronaut was set up to search the database of *H. longicornis* protein databases derived from transcriptome sequencing (NCBI accession number: GHLT00000000) (Wang et al., 2020) (19750 entries). Host *Oryctolagus cuniculus*, human keratin, and trypsin sequences were used as the contaminated database for proteomic searching. Trypsin was used as the digestion enzyme. Carbamidomethyl (C) was specified as the fixed modification. Oxidation (M), Acetyl (Protein N-term), Phospho (STY) were specified as the variable modifications. PTM localization filter was applied 0.75, and Qvalue (FDR) cutoff on precursor and protein level was applied 1%.

### Bioinformatics Analysis

Bioinformatics analysis was performed for all the phosphoproteins in saliva. PANTHER software (http://pantherdb.org/) was employed for the determination of Gene Ontology (GO) functional categories and protein classification. Pathways associated with the differentially expressed proteins were identified using the Kyoto Encyclopedia of Genes and Genomes (KEGG) database (http://www.kegg.jp/kegg/). KEGG figures were constructed using Omicsolution (https://www.omicsolution.org/wkomics/main/).

### RNA Interference

To perform RNA interference (RNAi), double-stranded RNA (dsRNA) was synthesized using the T7 RiboMAX™ Express RNAi System (Promega, USA) following the manufacturer’s guidelines. Specific *H. longicornis* nucleotide sequence targeting positions 1590-2008 of the SPK (GenBank: GHLT01011355), 4931-5317 of the TCP (GenBank: GHLT01016132), 227-630 of the PD (GenBank: GHLT01001774), and 72-420 of the ADF (GenBank: GHLT01011919), respectively, were selected for dsRNA synthesis. The target cDNAs were cloned and sequenced, followed by the synthesis of the double-stranded cDNAs containing T7 promoter (underlined) using the following primers: 5' - GGATCCTAATACGACTCACTATAGGACGGGGCAGACATCACC - 3' and 5'- GGATCCTAATACGACTCACTATAGG CGAAACGAACCTTCAAACC - 3' (Tudor domain-containing protein (TCP)); 5' - GGATCCTAATACGACTCACTATAGG TGGTTCGGGAATACGGT - 3' and 5' - GGATCCTAATACGACTCACTATAGG GTGTTGGCTTGGATGTGC - 3' (serine/threonine-protein kinase (SPK)); 5' - GGATCCTAATACGACTCACTATAGG CCCAGTACCGCAAGAACA - 3' and 5' - GGATCCTAATACGACTCACTATAGG CAGCAGCACGGAAGTCA - 3' (programmed cell death protein (PD)); 5' - GGATCCTAATACGACTCACTATAGG CCGCTACATCATCTACCACA - 3' and 5' - GGATCCTAATACGACTCACTATAGG CGACTCTATCGCCTCCTG - 3' (actin-depolymerizing factors (ADF)). For the control group, green fluorescent protein (GFP, GenBank: KX247384.1) dsRNA was synthesized. Twenty-five unfed female *H. longicornis* were injected with 0.5-1 μl (4 μg/μl) dsRNA using a Hamilton syringe (33-gauge needle) in the lower right quadrant of the tick (Kocan et al., 2009). After injection, the ticks were kept in an incubator (25 ± 1°C and 75% relative humidity) for 24 h to recover, then were allowed to feed on the ears of rabbits using cloth earmuffs. After the attachment/feeding period, the ticks were collected and mounted on the microscope, Axio Zoom V16 (Zeiss, Oberkochen, Germany) to observe for changes in phenotype.

The following parameters were used to evaluate the effect of the RNAi: (1) tick mortality rate; (2) time required for engorgement and weight of engorged tick; (3) the oviposition amount; and (4) egg hatching rate. These parameters were determined with the following formulae;


$$\text{Mortality rate}=\frac{number\;of\;dead\;ticks\;after\;RNAi}{number\;of\;ticks\;injected\;with\;dsRNA}$$

$$\text{Mean engorged weight}=\frac{total\;number\;of\;engorged\;female\;ticks}{number\;of\;engorged\;female\;ticks}\pm SD$$

$$\text{Mean number of eggs}=\frac{total\;number\;of\;eggs}{number\;of\;engorged\;female\;ticks}\pm SD$$

$$\text{Mean hatching rate}=\frac{total\;hatching\;number}{total\;egg\;number}\pm SD$$

$$SD\sqrt{\frac{1}{N}\sum_{i=1}^{N}{\left(x_{i}-u\right)}^{2}}u=\frac{1}{N}(x_{1}+\ldots \ldots x_{N})$$

Where SD is the standard deviation.

### RNAi Silencing Validation and Transcription Analysis of Target Genes

The degree of knockdown (RNAi silencing validation) of the four target genes was confirmed by evaluating mRNA expression levels in the salivary gland, ovary, and midgut using RT-qPCR after dissecting a separate set of dsRNA-injected ticks (unfed) in PBS (phosphate-buffered saline, 1 M) at pH 7.2, collection of the organs, flash-frozen in liquid nitrogen, and stored at –80°C until further analysis. Total RNA was extracted from the organs using TRIzol™ Reagent (Invitrogen, Carlsbad, CA), and then the concentration and purity of total RNA were assessed using NanoDrop 2000 (Thermo Fisher Scientific, Waltham, MA, USA). The complementary DNA (cDNA) was synthesized from 4 µg of total RNA by reverse transcription reaction using TransStart^®^ One-Step gDNA Removal and cDNA Synthesis SuperMix (TransGen Biotech, Beijing, China), following the manufacturer’s instructions. The mRNA expression of the target genes using cDNA was evaluated by RT-qPCR, which was performed *via* an Mx3005P qPCR system (Agilent Technologies, Santa Clara, USA) using TransStart^®^ Top Green qPCR SuperMix (TransGen Biotech, Beijing, China) according to the manufacturer’s guidelines. Actin was used as the reference gene and each sample was assessed in triplicate. The relative expressions of the target genes were calculated by 2^-ΔΔCT^ method (Livak and Schmittgen, 2001). RT-qPCR primers of the target genes are listed in **Supplementary Table S2**.

To determine whether the target genes were blood meal-induced or constitutively expressed, the mRNA expression of the target genes in semi-engorged ticks was compared to that of the unfed ticks, following the same RNA extraction, cDNA synthesis, and RT-qPCR protocols as previously described. Additionally, transcription analysis of *vitellin* in SPK-silenced ticks was performed *via* RT-qPCR to determine the effect of SPK on tick reproduction. *Vitellin* primers for RT-qPCR are shown in **Supplementary Table S3**.

To further investigate a possible effect of SPK on the rabbit immune system, proteomic analysis of rabbit plasma from the bite site by SPK silenced ticks were performed. The blood of 3 rabbits bitten by female ticks injected with GFP dsRNA (control group) and the blood of 3 rabbits bitten by female ticks injected with SPK dsRNA (treatment group) was drawn from the ear site, respectively. Heparin sodium salt was added for anticoagulation, centrifuged at 2000 g for 10 min, and the supernatant plasma was absorbed. An equal volume of pre-cooled tris-equilibrium phenol was added to the plasma. After shaking for 1 min at 12, 000 g, centrifugation at 4°C for 15 min, solution stratification appeared. The upper aqueous phase was removed. Then equal volume pre-cooled Tris-HCl (pH=8.0) was added and shaken for 1 min, then centrifuge at 12, 000 g at 4°C for 15 min. After removing the upper aqueous phase, the operation was repeated, then 5 times the volume of pre-cooled ammonium acetate methanol solution (0.1 M) was added, shaken for 1 min, and precipitated at −20°C overnight. Then the samples were centrifuged at 12, 000 g at 4°C for 15 min, and the upper liquid was discarded. 1 ml of pre-cooled methanol was added and mixed well *via* vortex vibration, then centrifuged at 12, 000 g at 4°C for 15 min, and the upper liquid was discarded. The sample was lyophilized and stored at −80°C for Data-independent acquisition (DIA) quantitative analyses.

### DIA Quantitative Proteomic Analysis

DIA quantitative analyses were performed using LC-MS with a UPLC M-Class system (Waters, USA) and a Q Exactive HF mass spectrometer (Thermo Fisher, USA). After loading each sample onto a C18 RP trap column (5 μm particle size, 100 Å pore size, 180 μm ID × 20 mm length; Waters, USA), they were separated on a C18 RP analytical column (1.8 μm particle size, 75 μm ID × 250 mm length; Waters, USA) at a flow rate of 300 nl/min with the linear ACN gradient as follows: 2-8% solvent B over 6 min and 8-35% solvent B over the next 114 min (solvent A: 99.9% H_2_O, 0.1% FA; solvent B: 99.9% ACN, 0.1% FA). The sample was electrosprayed into the Q Exactive HF mass spectrometer (voltage: 2.0 KV, heating capillary temperature: 290°C), and the parameters were set: DIA mode parameters of Q Extractive HF were set as follows: (a) scanning range was 350-1200 m/z; (b) resolution of the precursor ion was 60,000; (c) automatic gain control (AGC) target was 3×10^6^; (d) maximum ion injection time (maximum IT) was 50 ms; (e) 27% HCD normalized collision energy; (f) DIA method set as: Full MS (350 to 1250 m/z), followed by 20 DIA MSMS, and DIA isolation window (IW) were 59.0 m/z, 25.0 m/z, 19.0 m/z, 17.0 m/z, 13.0 m/z, 13.0 m/z, 11.0 m/z, 12.0 m/z, 12.0 m/z, 11.0 m/z, 12.0 m/z, 9.0 m/z, 10.0 m/z, 10.0 m/z, 11.0 m/z, 10.0 m/z, 10.0 m/z, 9.0 m/z, 10.0 m/z, 10.0 m/z; then Full MS (350 to 1250 m/z), followed by 20 DIA MSMS IW including 10.0 m/z, 9.0 m/z, 10.0 m/z, 8.0 m/z, 9.0 m/z, 9.0 m/z, 10.0 m/z, 10.0 m/z, 10.0 m/z, 10.0 m/z, 9.0 m/z, 10.0 m/z, 10.0 m/z, 10.0 m/z, 10.0 m/z, 10.0 m/z, 11.0 m/z, 10 m/z, 10 m/z, 11 m/z; then Full MS (350 to 1250 m/z), followed by 20 DIA MSMS IW including 10 m/z, 11 m/z, 12 m/z, 11 m/z, 13 m/z, 13 m/z, 13 m/z, 14 m/z, 13 m/z, 14 m/z, 14 m/z, 19 m/z, 18 m/z, 20 m/z, 27 m/z, 24 m/z, 33 m/z, 45 m/z, 56 m/z, 91 m/z. (g) MS2 scan resolution was 30,000, AGC target was 1×10^6^; raw data were analyzed using version 15.0 Spectronaut software (Switzerland), and using the default parameters for DIA data analysis (FDR<1%). The database of the *Oryctolagus cuniculus* (downloaded on 2021. 10. 10, contains 50426 sequences) was utilized for the DIA mass spectra searching. The selected search parameters were as follows: (i) trypsin digestion with 2 missed sites; (ii) N-terminal acetylation, variable modifications set methionine oxidation; (iii) fixed modifications set carbamidomethylation of cysteine. The proteomics data from the mass spectrometry was deposited to the ProteomeXchange Consortium (http://proteomecentral.proteomexchange.org) through the iProX partner repository (Project accession: PXD028182).

### Skin Biopsy at the Tick Bite Site and Microscopic Analysis

After local anaesthesia of rabbits, about 3 mm^3^ of the skin tissue of the normal ears and ears bitten by ticks were quickly removed with a scalpel and immediately placed in 1 ml of 10% formalin fixative in preparation for H&E staining and light microscopy observation. The skin tissues from the same part of the rabbit’s ear that was not bitten by a tick and the same part bitten by ticks injected with dsRNA-GFP were used as the control groups.

### Preparation of Paraffin Sections for H&E Staining and Observation

The paraffin sections for H&E staining and observation were prepared as described by Hu et al. (2021). Using a microtome (Leica RM2255, Germany), sections (3 mm^3^) were obtained from each paraffin block and stained with H&E. After the samples were immersed in xylene and alcohol, they were stained with haematoxylin for 15 min, followed by dehydration in 75% and 85% alcohol for 5 min each, and stained with eosin for 1 min, then re-immersed in ethanol and xylene. After the sections of the rabbit skin tissue were mounted in a synthetic resin (Entellan, Germany), they were observed and photographed under an optical microscope (Zeiss Imager A2, Germany).

## Results

**Figure 1:**
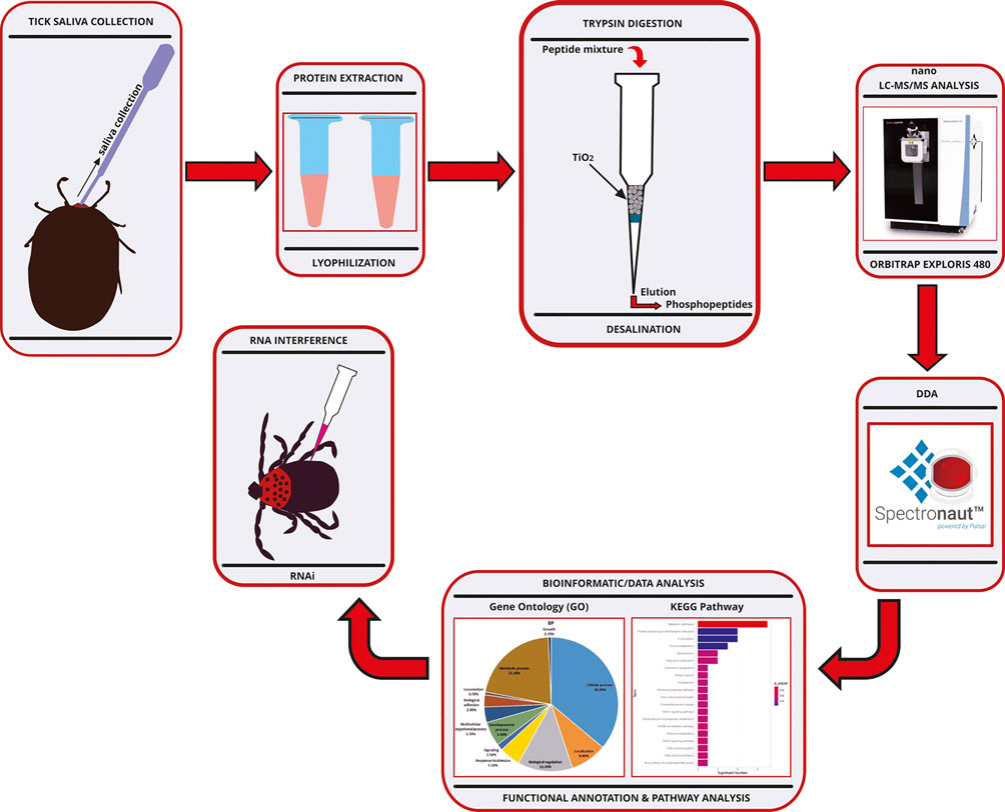
Experimental workflow chart for the phosphoproteomic analysis of the salivary proteins of *Haemaphysalis longicornis* tick. RP-HPLC, Reversed Phase-High Performance Liquid Chromatography; DIA, Data-Independent Acquisition.

The experimental workflow for the phosphoproteomic analysis of the salivary proteins of *Haemaphysalis longicornis* tick is shown in **Figure 1**. After the semi-engorged female ticks of *H. longicornis* were induced with dopamine, sufficient saliva was secreted and collected for analysis of the constituent phosphoproteomes. The phosphoproteomic analysis identified a total of 262 phosphorylated tick saliva proteins (**Supplementary Table S1**). The total ions chromatograph (TIC) and base peak chromatograph identified by mass spectrometry are shown in **Supplementary Figure S1**. The mass spectrometry proteomics data have been deposited in the ProteomeXchange Consortium (http://proteomecentral.proteomexchange.org) *via* the iProX partner repository (Project accession: PXD028182).

### Function Annotation of Phosphorylated Tick Saliva Proteome

**Figure 2:**
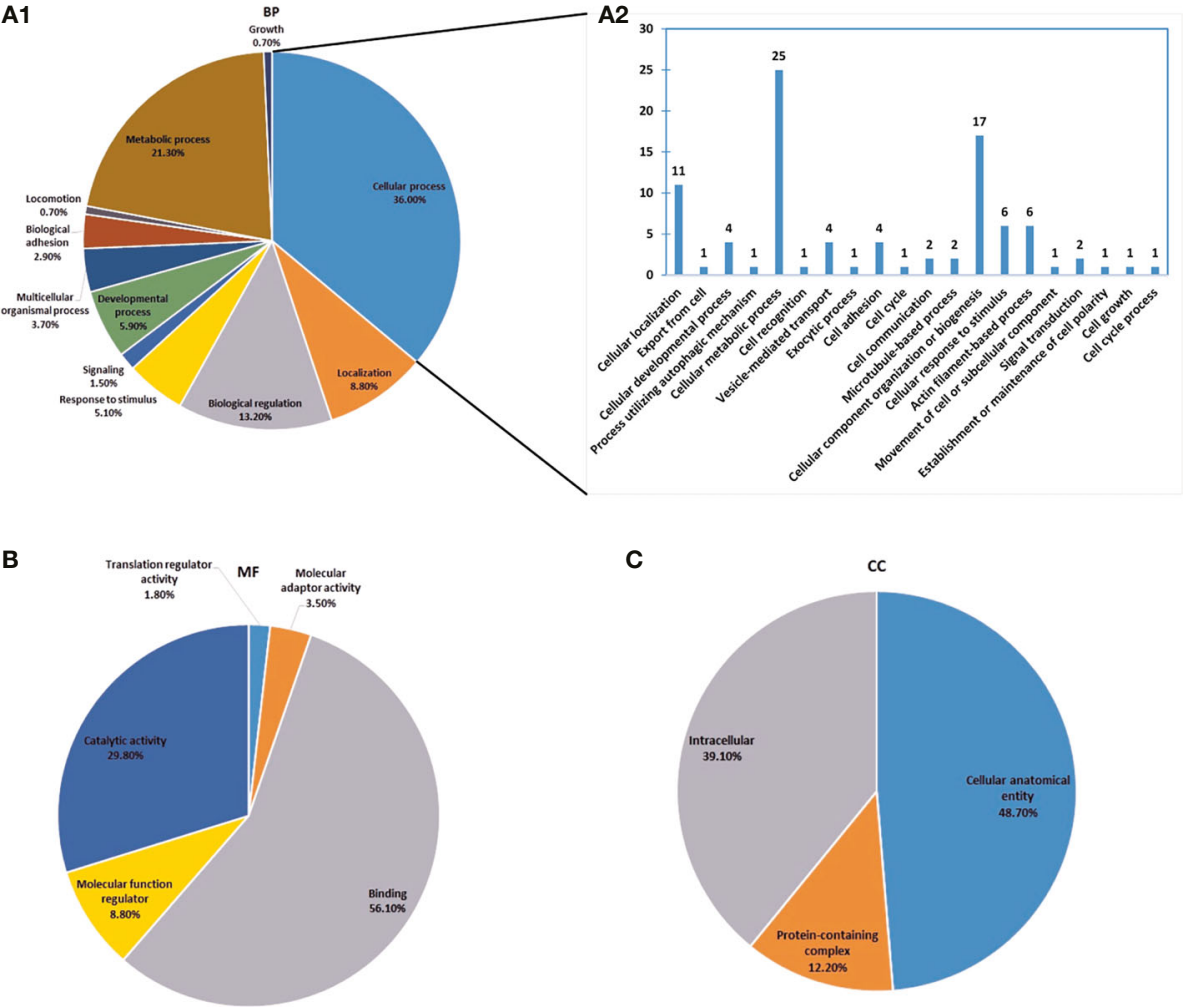
Gene Ontology (GO) analysis of phosphorylated proteins of *Haemaphysalis longicornis* tick saliva. The percentages of proteins assigned to the different terms are shown. **(A1, A2)** Biological process, with the expansion of the cellular process sector in A2. **(B)** Molecular function; **(C)** Cellular component.

Gene ontology (GO) enrichment analysis was performed to show the functional categorization of the phosphorylated tick saliva proteome. The classification of the proteins’ functional annotations was in three groups according to the GO enrichment analysis: biological process, molecular function, and cellular component. The functional categories associated with the phosphorylated tick saliva proteins were shown in **Figure 2**. A total of 11 terms were enriched in the biological process (BP) category, of which the cellular process term (36.0%) and metabolic process term (21.3%) accounted for the largest proportion of the saliva proteins. The cellular process had 20 subcategories of which comprises significant intracellular and extracellular transport-oriented processes such as vesicle-mediated transport, exocytic process, export from cell, cell adhesion, cell communication, movement of cell or subcellular component, and signal transduction, all of which play a vital role in facilitating blood acquisition and pathogen transmission during tick feeding. A total of 5 terms were enriched in the molecular function (MF) category. Among them, the binding term (56.10%) and catalytic activity term (29.80%) had the largest proportion of the saliva proteins, whereas, for the cellular component (CC) category, a total of 3 terms were enriched, of which the cellular anatomical entity term (48.70%) and the intracellular term (39.10%) accounted for the largest portion of the tick salivary proteins.

The proteins were classified into 13 groups according to PANTHER protein classification (**Figure 4**). The cytoskeletal protein, metabolite interconversion enzyme, nucleic acid metabolism protein, and scaffold/adaptor protein classes were comprised of the largest number of proteins.

### KEGG Pathway Analysis of Phosphorylated Tick Salivary Proteome

**Figure 3:**
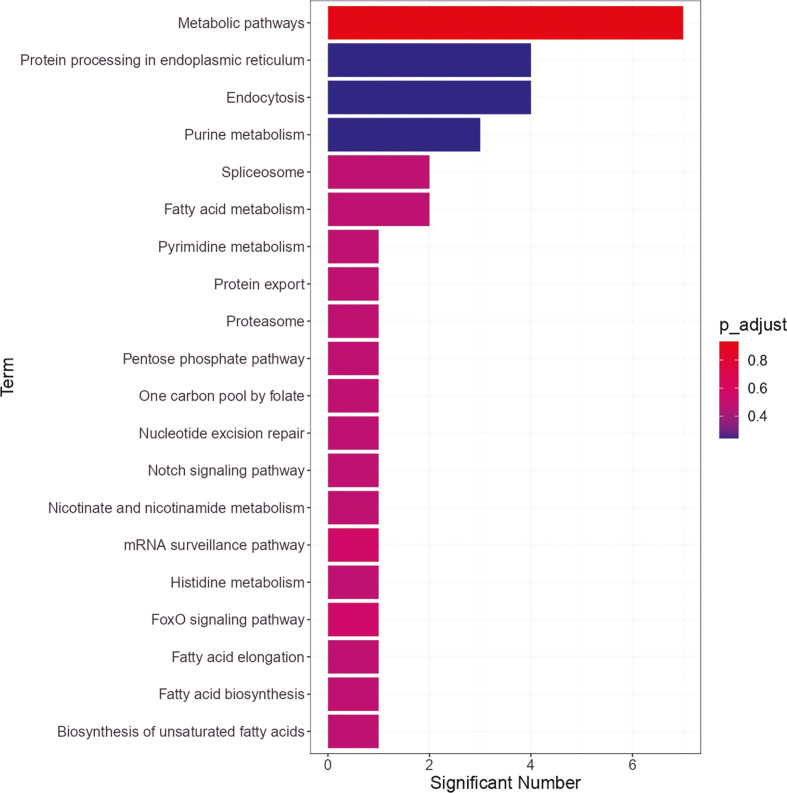
Enriched Kyoto Encyclopaedia of Genes and Genomes (KEGG) pathways of the phosphoproteins of *Haemaphysalis longicornis* tick saliva.

The phosphorylated tick salivary proteome was subjected to KEGG pathway enrichment analysis (**Figure 3**). “Endocytosis”, “Protein processing in endoplasmic reticulum”, and “Purine metabolism” were the most significantly enriched pathways. Dynamin, H (beta) 58 protein, vacuolar sorting protein, and sorting nexin were associated with the “Endocytosis” pathway. Heat shock HSP20 protein, UBX domain-containing protein, and nucleotide excision repair factor NEF2, RAD23 component participated in the “Protein processing in endoplasmic reticulum”, whereas, GARS/AIRS/GART was associated with “Purine metabolism” pathway.

### Protein Classification (PANTHER)

The phosphorylated proteins were grouped into classes according to PANTHER protein classification. A total of 13 PANTHER protein classes were annotated (**Table 1**). The cytoskeletal protein (PC00085), metabolite interconversion enzyme (PC00262), nucleic acid metabolism protein (PC00171), scaffold/adaptor protein (PC00226), and protein modifying enzyme (PC00260) were the most enriched classes.

**Table 1:** Categorization of the phosphorylated saliva proteins of *Haemaphysalis longicornis* ticks according to PANTHER protein classification.

Protein classification (PANTHER)	Phosphorylated proteins with UNIPROT accession numbers	Number/percentage of proteins
Protein modifying enzyme (PC00260)	Ubiquitin specific peptidase, putative (B7P8E0); Proteasome subunit alpha type (B7PNN1); Protein kinase domain-containing protein (B7PZ91); Pole hole family protein (B7Q903); hypothetical protein (B7QLS5).	5 (8.3%)
Chromatin/chromatin-binding, or -regulatory protein (PC00077)	DEK_C domain-containing protein (B7P839); apoptotic protease-activating factor, putative (B7P8X7); DNA replication factor/protein phosphatase inhibitor SET/SPR-2, putative (B7Q573)	3 (5%)
Cytoskeletal protein (PC00085)	SH3 domain-containing protein (B7P0V4); Aldolase_II domain-containing protein (B7P1C8); PDZ domain-containing protein (B7P6T6); Microtubule-binding protein, putative (B7PPL3); LIM zinc-binding domain-containing protein (B7PU84); Microtubule-associated protein 1S, putative (B7PZF5); Paxillin, putative (B7Q0A4); Uncharacterized protein (B7Q121); Tensin, putative (B7Q615); 65-kDa macrophage protein, putative (B7QBZ0).	10 (16.7%)
Extracellular matrix protein (PC00102)	SPARC_Ca_bdg domain-containing protein or microtubule-associated protein 1S, putative (B7QDS2)	1 (1.7%)
Gene-specific transcriptional regulator (PC00264)	MYCBP, AMY1: C-Myc-binding protein, putative (B7P219); C2H2-type domain-containing protein (B7P493); Woc protein, putative (B7PYR4).	3 (5%)
Membrane traffic protein (PC00150)	Vacuolar protein-sorting protein, putative (B7P4N4); Dynamin GTPase (B7PM12); H(Beta)58 protein, putative (B7PQ07); Vacuolar sorting protein, putative (B7QLI1)	4 (6.7)
Metabolite interconversion enzyme (PC00262)	Ribokinase (B7P0U8); 5’ nucleotidase, putative (B7P150); Adenosine diphosphatase, putative (B7PXK0); Pribosyltran_N domain-containing protein (B7PYZ2); Histidine ammonia-lyase (B7QBL3); Superoxide dismutase [Cu-Zn] (B7QEW8); Nudix hydrolase domain-containing protein (B7QFY3); Short chain alcohol dehydrogenase, putative (B7QMN2); N-acetyltransferase domain-containing protein (B7QNQ0).	9 (15%)
Nucleic acid metabolism protein (PC00171)	Mid1-interacting protein, putative (B7P1V6); RNA helicase, putative (B7PAK3); AAR2 splicing factor homolog (B7PMS5); RRM domain-containing protein (B7PWM5); ATP-dependent RNA helicase, putative (B7Q1P2); Poly(A) polymerase (B7Q2V7); General vesicular transport factor p115-like isoform X1 (B7Q3I5); UV excision repair protein RAD23 (B7Q760); PWI domain-containing protein (B7QAA4).	9 (15%)
Protein-binding activity modulator (PC00095)	Dock-1, putative (B7P9N2); NSFL1 cofactor p47 (B7PEL3); Rab-GAP TBC domain-containing protein (B7PEM0); CYCLIN domain-containing protein (B7Q1T0)	4 (6.7%)
Scaffold/adaptor protein (PC00226)	SH3 domain-containing protein (B7PEH9); Leucine rich domain-containing protein, putative (B7PV81); FHA domain-containing protein (B7Q7N2); Kinectin, putative (B7Q7V6); PX domain-containing protein (B7Q8P2); Fyn-binding protein, putative (B7Q9D2); Tudor domain-containing protein, putative (B7QEM7); Secreted protein, putative (B7QIE6).	8 (13.3%)
Transfer/carrier protein (PC00219)	Uncharacterized protein (B7P7Q7)	1 (1.7%)
Translational protein (PC00263)	Programmed cell death protein 4 (B7PJK8); PUA domain-containing protein (B7PS91).	2 (3.3%)
Transmembrane signal receptor (PC00197)	Cytochrome b5 heme-binding domain-containing protein (B7PN29)	1 (1.7%)

### RNAi Silencing Validation And Transcription Analysis of Target Genes/Proteins

**Figure 4:**
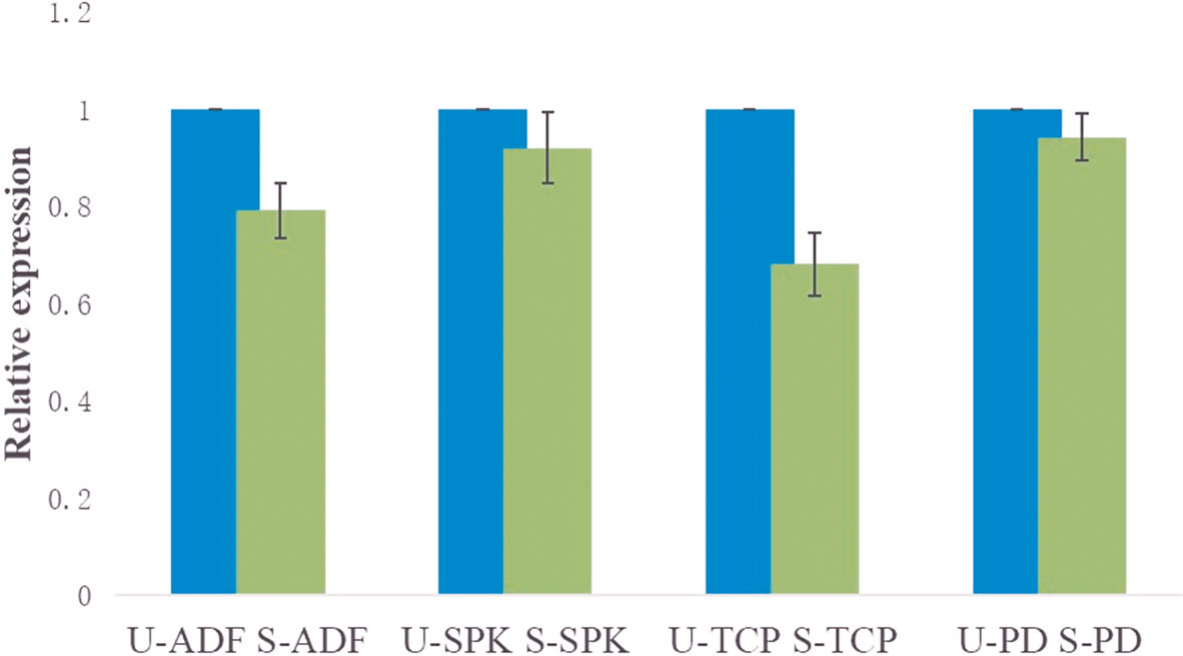
Transcription analysis of *ADF*, *SPK*, *TCP*, and *PD* in semi-engorged ticks relative to the unfed ticks evaluated *via* RT-qPCR. The results are expressed as the means (n = 3) ± SEM. ADF, actin-depolymerizing factors; SPK, Serine/threonine-protein kinase; TCP, Tudor domain-containing protein; and PD, Programmed cell death protein; U-, unfed; S-, semi-engorged.

**Figure 5:**
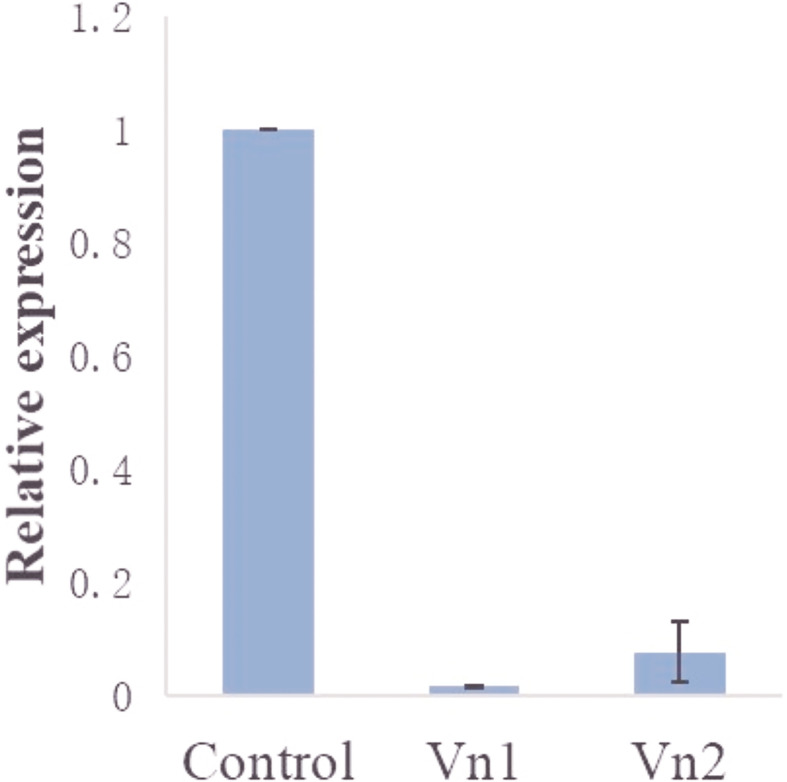
Expression of *vitellin 1* (Vn1) and *vitellin 2* (Vn2) in SPK-silenced ticks relative to the control (dsRNA-GFP-injected group). The results are expressed as the means (n = 3) ± SEM.

The mRNA expression of the target genes was low after gene knockdown relative to the control group, which demonstrates the efficiency of the RNAi (**Supplementary Figure S2**). The transcription analysis of *ADF*, *SPK*, *TCP*, and *PD* in semi-engorged ticks showed a slightly lower mRNA expression of the target genes relative to the unfed ticks, which was insignificant and indicates that the target genes were constitutively expressed and not induced by blood meal (**Figure 4**). In addition, the expression of *vitellin 1* and *vitellin 2* in SPK-silenced ticks was very low relative to the control group (**Figure 5**), demonstrating the effect of SPK on tick reproduction.

Proteomic analysis of rabbit plasma from the bite site by SPK silenced ticks to further investigate a possible effect of SPK on the rabbit immune system, identified some upregulated and downregulated immune-related proteins (**Supplementary Table S4**).

### Changes in Tick Phenotype After RNAi

**Figure 6:**
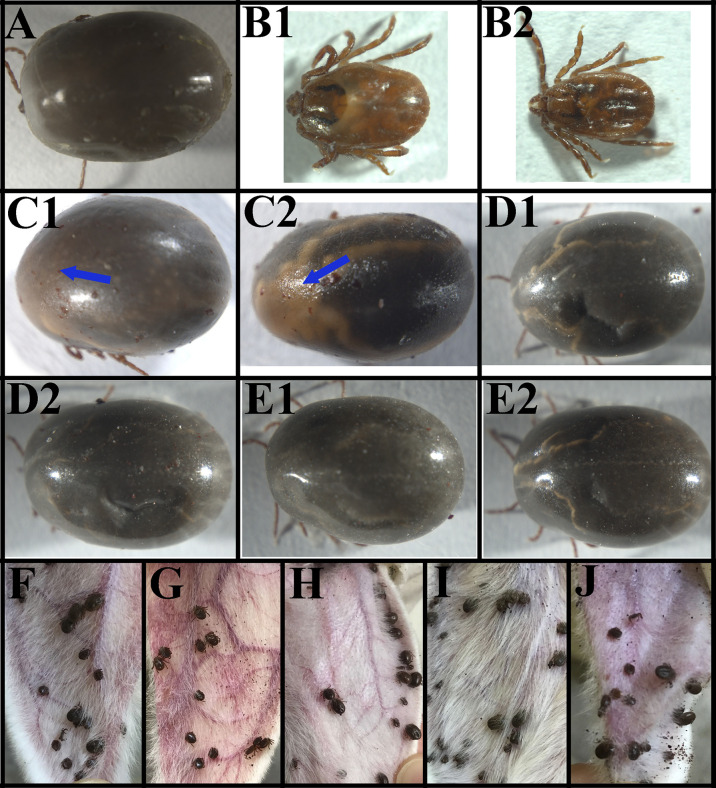
The phenotype associated with actin-depolymerizing factors (ADF), serine/threonine-protein kinase (SPK), Tudor domain-containing protein (TCP), and programmed cell death protein (PD) mRNAs subjected to RNAi in female ticks *via* injection with the corresponding dsRNAs. **(A)** Ticks after 7 days of GFP dsRNA injection (control); **(B1, B2)** Ticks after 7 days of ADF dsRNA injection, note the inability of the ticks to engorge; **(C1, C2)** Ticks after 7 days of SPK dsRNA injection; note the change in skin colour; **(D1, D2)** Ticks after 7 days of TCP dsRNA injection (no changes were observed); **(E1, E2)** Ticks after 7 days of PD dsRNA injection (no changes were observed); **(F)** Ticks injected with GFP dsRNA (4th day) (control); **(G)** Ticks injected with ADF dsRNA (4th day), note that they could not engorge; **(H)** Ticks injected with PD dsRNA (4th day); **(I)** Ticks injected with SPK dsRNA (4th day); **(J)** Ticks injected with TCP dsRNA (4th day).

**Supplementary Table S5** showed the number of ticks that attached and fed to repletion from the total number of ticks thrown on the rabbit host after RNAi of the ticks. **Figure 6A** shows the GFP dsRNA-injected (control) group 7 days post-injection. Observable changes occurred 7 days post-injection of actin-depolymerizing factor (ADF) dsRNA (**Figures 6B1, B2**) and serine/threonine-protein kinase (SPK) dsRNA (**Figures 6C1, C2**). The *H. longicornis* ticks injected with the dsRNA of ADF failed to engorge after seven days of attachment to the rabbit’s ear (**Figures 6B1, B2**), unlike the GFP dsRNA-injected group (control), underscoring the role of ADF in the facilitation of tick feeding. The ticks subjected to SPK knockdown engorged normally but with a change in skin colour (possibly an autoimmune reaction) and failure to produce eggs (**Figures 6C1, C2**), unlike in the control group injected with GFP dsRNA (**Figure 6A**) pointing to a possible role of SPK in tick reproduction and host immune modulation. No observable changes were observed on the ticks after 7 days of TCP (**Figures 6D1, D2**) and PD dsRNA injections (**Figures 6E1, E2**). After 4 days of attachment to the rabbits’ ear, the ticks injected with GFP dsRNA (**Figure 6F**), PD dsRNA (**Figure 6H**), SPK dsRNA (**Figure 6I**), and TCP dsRNA (**Figure 6J**) had commenced engorgement, apart from the ADF dsRNA-injected group of ticks (**Figure 6G**), which did not have a successful blood meal. The summary of effects of the RNAi on engorgement time, mortality rate, tick weight, number of eggs, and egg hatching rate of *Haemaphysalis longicornis* ticks is shown in **Table 2**.

**Table 2:** The effects of the RNAi on engorgement time, mortality rate, tick weight, number of eggs, and egg hatching rate of *Haemaphysalis longicornis* ticks.

	The mean time required for engorgement (days)^a^	Mortality rate (%)	Mean engorged weight (mg)^a^	Mean number of eggs^a^	Mean egg hatching rate (%)^a^
SPK	5.9 ± 0.8	33.3	106.4 ± 46.1	0	0
PD	6.5 ± 0.6	43.3	103.1 ± 47.1	1287.2 ± 637.1	61.5 ± 53.7
TCP	6.2 ± 0.7	46.6	126.2 ± 40.4	1484.8 ± 577.3	74.9 ± 19.7
ADF	0	51.1	0	0	0
GFP	5.2 ± 0.5	26.6	117.3 ± 39.8	1507.1 ± 577.1	86.3 ± 27.0
No injection	5.1 ± 0.5	15.1	124.0 ± 23.3	1878.0 ± 45.0	89.1 ± 14.0

a Mean ± standard deviation (SD).

### Microscopic Investigation of the Bite Site From Skin Biopsy

**Figure 7:**
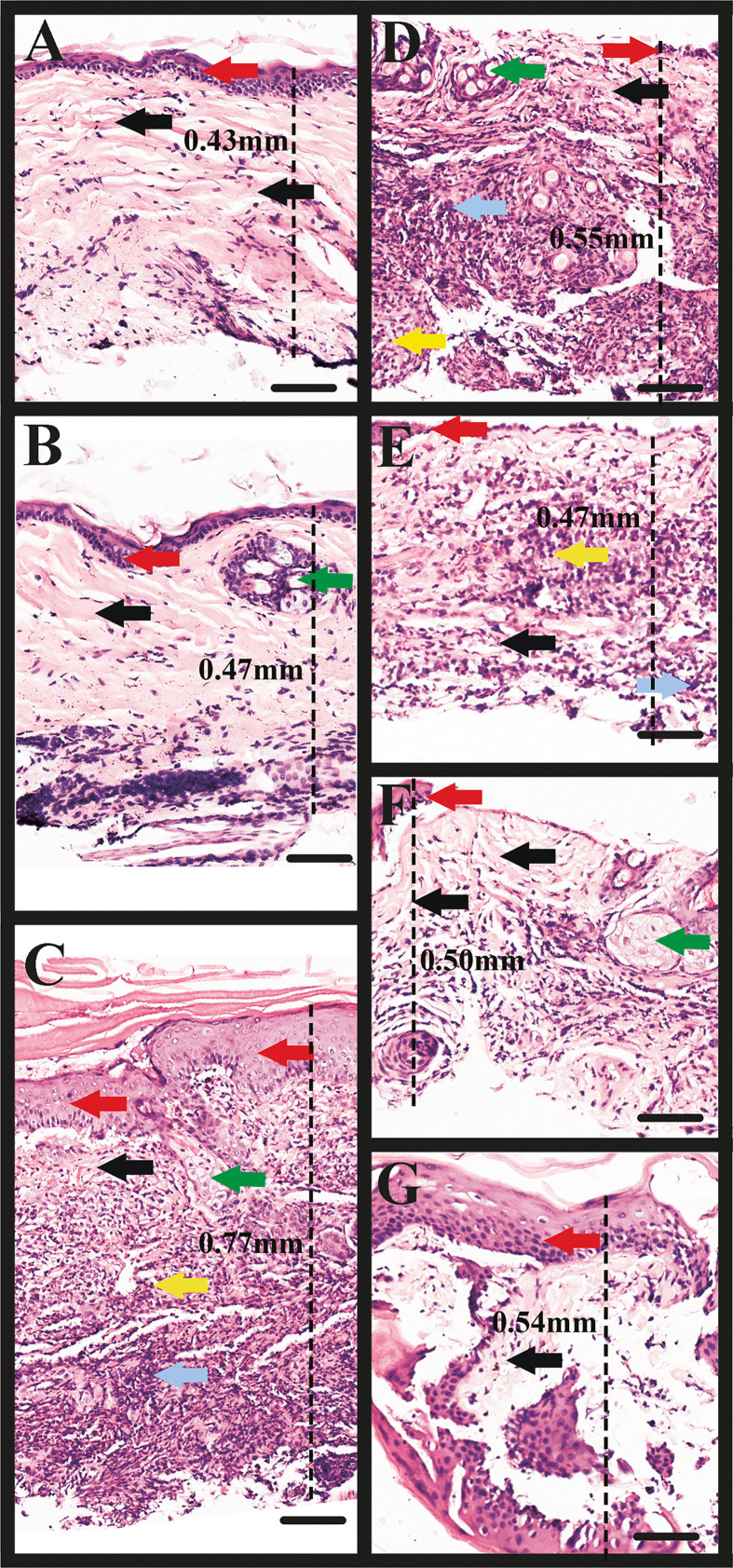
Microscopic image of rabbit skin. **(A, B)** Control group: the black and red arrows indicate the integrity of the collagen fibers and epithelium, respectively; the green arrow shows the pilosebaceous unit (the hair follicle and sebaceous gland). **(C)** Rabbit skin site (ear) bitten by the control ticks (GFP dsRNA-injected). Note the dissociation of collagen fibers (black arrows), and the inflammation of the epithelium (red arrows), inflammatory infiltrate/granulocytes (yellow arrow), haemorrhage (grey arrow), and part of the pilosebaceous unit (green arrow). **(D)** Rabbit skin site (ear) bitten by TCP-silenced ticks. Note the concentration of inflammatory infiltrate/granulocytes, predominantly heterophiles/neutrophiles (yellow arrow), haemorrhage (grey arrow), the dissociation of fibers (black arrow), disruption of the epithelium (red arrow), and pilosebaceous unit (green arrow). **(E)** Rabbit skin site (ear) bitten by ADF-silenced ticks. Observe the inflammatory infiltrate/granulocytes (yellow arrow), haemorrhage (grey arrow), dissociation of fibers (black arrow), and depletion of the epithelium (red arrow). **(F)** Rabbit skin site (ear) bitten by PD-silenced ticks. Observe the less dense dermis and dissociated fibers (black arrows), disrupted epithelium (red arrow), pilosebaceous unit (green arrow). **(G)** Rabbit skin site (ear) bitten by SPK-silenced ticks injected with serine/threonine-protein kinase (SPK) dsRNA (SPK knockdown). Note the disorganized dermis and fibers (black arrow), and inflamed epithelium (red arrow). Observe the increase in skin thickness (increased distance between the epidermis and the muscle/subcutaneous layer) as measured in the microscopic images **(C–G)**, which characterizes oedema. TCP, Tudor domain-containing protein; ADF, Actin-depolymerizing factors; PD, Programmed cell death protein; and SPK, Serine/threonine-protein kinase. Scale bars: 100 µm.

**Figures 7A, B** represent the control group (without tick bite); the black and red arrows indicate the integrity of the collagen fibers and epithelium, respectively, while the green arrow shows the pilosebaceous unit (the hair follicle and sebaceous gland). A dissociation of collagen fibers and cells in dermal layers was observed in all the experimental groups coupled with an increased skin thickness (increased distance between the epidermis and the muscle/subcutaneous layer) as measured in the microscopic images (**Figures 7C–G**), which characterizes oedema (measurements of skin thickness of all the groups are shown in **Table 3**). Inflammatory infiltrates/granulocytes (yellow arrow), which could be predominantly heterophiles/neutrophiles, were observed in **Figures 7C–E**. Depleted and disrupted epithelium were observed in all the experimental groups (**Figures 7D–G**), except for the site bitten by the control ticks (GFP dsRNA-injected) where the epithelial layer was inflamed (**Figure 7C**). Unlike the site bitten by the control ticks (GFP dsRNA-injected), most of the experimental groups exhibited the disorganization of the dermal structures and the pilosebaceous unit (**Figures 7D–G**) underscoring the importance of the tick saliva proteins in maintaining an orderly and conducive atmosphere for a successful blood acquisition to take place. An intense haemorrhage/extravasated blood (blue arrow) was observed, especially in **Figures 7C, D**, indicating the pool-feeding nature of ticks. The disorganization of the epidermis and dermis (**Figures 7C–G**) of the bite site showed the disruptive nature of the tick’s piercing of the skin with the hypostome at the feeding site.

**Table 3:** Measurements of rabbit skin thickness indicating the extent of oedema at the tick bite site relative to the control (no tick bite).

Group	Type of exposure	Measurement of skin thickness
Figure A (Control)	No tick bite	0.43 mm
Figure B (Control)	No tick bite	0.47 mm
Figure C	dsRNA-GFP tick bite site	0.77 mm
Figure D	dsRNA-TCP tick bite site	0.55 mm
Figure E	dsRNA-ADF tick bite site	0.47 mm
Figure F	dsRNA-PD tick bite site	0.50 mm
Figure G	dsRNA-SPK tick bite site	0.54 mm

## Discussion

### The Proteome of *H. longicornis* Tick Saliva

Previous studies have identified several tick saliva proteins that play vital roles during tick feeding but, to the best of our knowledge, no investigation has been carried out on the phosphorylated tick saliva proteins. Owing to the importance of phosphorylation in different cellular processes, the present study investigated the phosphorylated saliva proteomes of the tick *H. longicornis*. It is noteworthy that molecules and proteins of tick saliva exhibit functional redundancy — more than one tick salivary molecule could target the same host cell population; and pluripotency — a single salivary molecule may influence more than one host cell population (Chmelař et al., 2016; Aounallah et al., 2020). Thus, the functions of several phosphorylated proteins identified in this study could overlap in different ways. Additionally, given that ticks can recycle the host proteins in their saliva (Oliveira et al., 2013), this study screened out the host (rabbit) proteins using the database repertoire of rabbit proteins. However, a couple of the identified proteins which seem to belong to the host (rabbit) were highly conserved and showed high similarity to tick proteins which was also the case in previous studies (Oliveira et al., 2013; Tirloni et al., 2015). Thus, they could have originated from *H. longicornis* and not from the host.

Notably, previous studies have made some interesting hypotheses regarding the presence of host proteins in tick saliva. The movement of intact dietary and xenobiotic proteins (without modification) across the digestive system to the haemolymph has been demonstrated by different haematophagous and non-haematophagous insects (Jeffers and Michael Roe, 2008). It was suggested that these proteins may be transported from the haemolymph and digestive system to the salivary glands where they are secreted back into the host (Wang and Nuttall, 1994; Valenzuela et al., 2000; Madden et al., 2002; Francischetti et al., 2011). Other possible explanations of the presence of the host proteins in tick saliva indicated that the ticks recycle key host proteins in order to undermine their functions in the host (Carvalho et al., 2010); and that the host proteins could pass through the tick glycosylation machinery and be presented back to the host as immunogenic sugar-based antigens that could play a decoy role to divert the host immunity away from the tick proteins (Oliveira et al., 2013).

### Protein Phosphorylation Plays a Significant Role During Tick Blood Acquisition

Protein phosphorylation is a vital mechanism of regulation that influences most cellular processes including signal transduction, protein synthesis, cell division, cell growth, and development (Ardito et al., 2017). Kinases and phosphatases determine phosphorylation and dephosphorylation events which activate/deactivate many enzymes and receptors (Mccance and Huether, 2014). In the present study, 262 salivary proteins of *H. longicornis* were phosphorylated. Although most of the proteins were functionally annotated and enriched in GO terms, a significant number still appear to be functionally redundant given that their specific individual contribution to successful blood acquisition and pathogen transmission during tick feeding is not clear. It could be that most of these phosphorylated proteins have to work synergistically to exert some sort of influence during blood meal acquisition. A sizeable number can be categorized as housekeeping proteins, whereas, the most relevant proteins that facilitate tick feeding and pathogen transmission can be grouped as transporters (Francischetti et al., 2009; Bensaoud et al., 2019). This strongly corroborates the functional annotation where relevant phosphorylated proteins were functionally enriched under GO terms that have to do with different forms of intracellular and extracellular transport such as vesicle-mediated transport, exocytic process, export from cell, cell adhesion, cell communication, movement of cell or subcellular component, and signal transduction.

Some relevant proteins such as proteophosphoglycan, serine/threonine-protein kinase, phosphoserine phosphatase, among others, have 100 percent phosphorylation sites within their amino acid sequences. An interesting type of protein modification known as protein phosphoglycosylation has been observed to be predominant in protozoan parasites such as *Leishmania mexicana*, *Trypanosoma cruzi*, and *Entamoeba histolytica* inhabiting several vectors (Ilg, 2000; Moody-Haupt et al., 2000; Majumdar et al., 2005; Allen et al., 2013). The proteophosphoglycan identified in the saliva of *H. longicornis* in the present study is a glycoprotein that contains oligosaccharides linked to serine or threonine moieties through a phosphodiester linkage which is a result of protein phosphoglycosylation (Majumdar et al., 2005). The proteophosphoglycan in *H. longicornis* saliva could have been secreted by the protozoan parasites inhabiting the tick salivary gland. Many cellular proteins of eukaryotes depend on the covalent attachment to sugar units which confer on them a broad range of crucial biological functions including protection from proteolytic attack, immunogenicity, cell-cell communication, induction and maintenance of the protein conformation in biologically active forms, and solubility (Lis and Sharon, 1993; Dwek, 1995; Majumdar et al., 2005). Notably, part of the immune response of the host body during tick feeding is the release of antibodies and enzymes to proteolytically digest the antigens in the salivary constituent. We speculate that the functional implication of the proteophosphoglycan is to counter the proteolytic attack from the host and ensure that the conformation of the salivary proteins/peptides remain in their biologically active forms. It could also be a form of strategic immunity of the protozoan parasites against the host antibodies. Further studies are required to verify these hypotheses.

Unlike in ticks where the function of proteophosphoglycan is still relatively unknown, the role of proteophosphoglycan during blood acquisition has been reported in the sand fly (Giraud et al., 2018). Proteophosphoglycans from *Leishmania* which are secreted in the midgut of the sand fly form a biological plug called the promastigote secretory gel (PSG). The PSG blocks the gut of the sand fly and then induces the regurgitation of infective parasites, as well as facilitates dermal wound repair (Giraud et al., 2018). Similar activities of proteophosphoglycans could apply to ticks given that sand flies are also haematophagous arthropods.

### Serine/Threonine-Protein Kinase Could Act as a Molecular Switch That Enhances Functional Redundancy and Pluripotency in Salivary Proteins

In this study, serine/threonine-protein kinase (SPK) was identified in *H. longicornis* saliva and this could constitute substantial functional implications given the key role of protein kinases (PTKs) in diverse biological activities. Serine/threonine-protein kinase is a type of PTK that orchestrates the regulation of proteins’ biological activities by phosphorylating the hydroxy group of serine and threonine, thus triggering a conformational change from an inactive to an active form of a protein (Avendaño and Menéndez, 2008). Of note, many salivary peptides/proteins could affect the same host cell population (functional redundancy), and likewise, one salivary peptide/protein could target more than one host cell population (pluripotency) (Aounallah et al., 2020), and these activities could be championed by the presence of serine/threonine-protein kinase.

Posttranslational modifications (phosphorylation) are reversible, and SPK could act as a molecular switch that activates the functions of several salivary proteins, whereas, phosphoserine phosphatase (also identified in the saliva) could mediate the deactivation (switch off) of their functions (Chiba et al., 2012). In the present study, after the ticks were injected with SPK dsRNA (RNAi), they engorged normally but failed to produce eggs unlike in the control group injected with GFP. This result indicates a possible role of SPK in tick reproduction, which strongly corroborates its role during embryonic development in *Xenopus laevis* (Su et al., 1996). Also, the skin of the engorged ticks significantly changed in colour. A possible explanation could be that the immune cells of the rabbit’s blood were not sufficiently suppressed in the absence of SPK in the tick saliva resulting in an autoimmune reaction that manifested on the tick’s skin, given the role of SPK in switching on some host immune-modulatory proteins in the saliva. Phosphorylation activity mediated by SPK may be required to fully activate the functions of some tick proteins. The proteomic analysis of rabbit plasma from the bite site by SPK silenced ticks to further investigate a possible immune-regulatory role of SPK identified some upregulated and downregulated immune-related proteins. However, it is not clear exactly how SPK exerts influence on the rabbit immune-related proteins given the large number of differentially expressed proteins identified. In addition, substantial disorganization of the dermal structures and the pilosebaceous unit were observed at the rabbit’s skin site bitten by SPK silenced ticks. Further studies are required to fully grasp the functional implications of SPK during tick feeding and reproduction.

### Proteins Transfer and Pathogen Transmission During Tick Feeding Are Mediated by Extracellular Vesicles and Exosomes

There is a growing consensus among tick/arthropod vector researchers that the transmission of pathogens and vital molecules to the host during tick feeding is mediated by extracellular vesicles and exosomes (Raab-Traub and Dittmer, 2017; Hackenberg and Kotsyfakis, 2018; Zhou et al., 2018; Nuttall, 2019; Oliva Chávez et al., 2021). In the present study, the “Endocytosis” (KEGG) pathway was enriched with dynamin, sorting nexin, vacuolar sorting protein, and H (beta) 58 protein. These proteins were also associated with the “Vesicle-mediated transport” of the “Biological process” (GO term). Notably, the saliva-induced modulation of host responses using the salivary molecules *via* mainly the vesicle-mediated transport is being exploited by tick-borne pathogens in what is known as saliva-assisted transmission (SAT) (Hackenberg and Kotsyfakis, 2018; Nuttall, 2019). Extracellular vesicles (EVs) that are released from the multivesicular bodies (MVBs) are called exosomes (Raab-Traub and Dittmer, 2017). It has been long known that malignant and virus-infected cells release vesicles but the fact that perfectly healthy cells also secrete vesicles has only been appreciated recently (Hristov et al., 2004). EVs act as vehicles for transfer between cells of membrane and cytosolic proteins, RNA, and lipids, and thus, are crucial modes of intercellular communication (Raposo and Stoorvogel, 2013).

Dynamin, one of the phosphorylated *H. longicornis* salivary proteins that participated in the vesicle-mediated transport and endocytosis pathway, was previously identified in the salivary gland of *H. longicornis* (Xiao et al., 2020). Dynamin is a GTPase that is a vital component of vesicle formation in receptor-mediated endocytosis and plays a significant role in caveolae internalization, synaptic vesicle recycling, and Golgi-associated vesicle trafficking (Hinshaw, 2000). The hypothesis that dynamin plays a strategic role in membrane fission by wrapping around the necks of budding vesicles (Hinshaw, 2000) suggests that it gives extra mechanoprotection to the vesicles to be delivered to the host from the tick saliva. This ensures that the bioactive molecular packages to be delivered to the host *via* the saliva during blood acquisition remain intact. Additionally, it could be that pathogens take advantage of this extra layer of protection provided by dynamin for their safe transmission to the host. Further studies are thus required to characterize their functions in the extracellular environment.

Sorting nexin, another identified phosphorylated protein in the saliva of *H. longicornis* that participated in the vesicle-mediated transport and endocytosis pathways, were characterized in previous studies as a wide-ranging group of cytoplasmic proteins connected by a phospholipid-binding motif (PX domain) and are involved in membrane trafficking and protein sorting (Bravo et al., 2001; Worby and Dixon, 2002). They have a special ability to bind phospholipids, form protein-protein complexes, and facilitate protein sorting (Worby and Dixon, 2002). Although its function during tick feeding is undefined, it could be that its presence in the tick saliva modulates the host proteins capable of jeopardizing the feeding process, given that its primary function is to regulate membrane trafficking and protein-protein interaction. Thus, more studies are needed to verify its main function during tick feeding.

### The Vasodilator-Stimulated Phosphoprotein Plays a Key Role in the Actin-Based Movement of Pathogens During Tick Feeding

Vasodilator-stimulated phosphoprotein (VASP), a family of proteins with a highly conserved structure, was identified in the saliva of *H. longicornis* in the present study. VASP has been identified on the F-actin tail and/or on surfaces of several intracellular pathogens such as *Rickettsia*, *Vaccinia virus*, and *Shigella flexneri* (Chakraborty et al., 1995; Frischknecht et al., 1999; Van Kirk et al., 2000). Of note, VASP proteins may perform different roles depending on the cellular context (Loureiro et al., 2002). We hypothesize that during blood acquisition by *H. longicornis*, the presence of VASP in the tick saliva could increase the speed of pathogens and enhance their navigation from cell to cell, thus, facilitating pathogenicity. VASP structure comprises an N-terminal EVH1 domain, a central proline-rich region, and a C-terminal EVH2 domain (Wentworth et al., 2006). When they invade the mammalian cells, viruses and bacteria such as *Listeria monocytogenes* employ the host cell’s actin cytoskeleton for movement within and from cell to cell (Krause et al., 2003). *Listeria* has a bacterial surface protein known as ActA (consists of proline-rich repeats) which is vital for its intracellular motility (Domann et al., 1992; Kocks et al., 1992). VASP uses its EVH1 domain to directly bind to these proline-rich repeats (a ligand for EVH1 domain) within ActA, and this complex regulates actin dynamics (Chakraborty et al., 1995; Niebuhr et al., 1997; Krause et al., 2003). An experiment in which the proline-rich repeats within ActA were deleted revealed a reduction of intracellular speed of the bacteria and pathogenicity (Lasa et al., 1997; Niebuhr et al., 1997). Interestingly, VASP proteins are one of the substrates for serine/threonine kinases (also identified in *H. longicornis* saliva) which modulates their functions within cells *via* phosphorylation (Krause et al., 2003).

### Tudor Domain-Containing Proteins in Tick Saliva May Be Associated With the Modulation of Host Immune Proteins

Tudor domain-containing protein (TCP) was identified in *H. longicornis* saliva in the present study and this could have significant functional implications during tick feeding. Originally, the Tudor domain was identified in Tudor protein encoded in *Drosophila* as a well-conserved protein structural domain (Botuyan and Mer, 2016). They act as adaptor proteins and can alter transcription by involving in epigenetic regulation *via* the recognition of post-translational histone modification (Lu and Wang, 2013). This definition strongly supports TCP in silico categorization as “scaffold/adaptor protein” in PANTHER protein classification. Tudor proteins employ DNA-methyltransferases to suppress transcription *via* the facilitation of DNA methylation and heterochromatin assembly (Botuyan and Mer, 2016). During tick feeding, Tudor domain-containing proteins are injected into the host blood *via* the saliva, and apparently, the resultant effect is the disruption of the epigenetic landscapes which have a significant impact on the immune homeostasis of the host, owing to the indispensability of epigenetic factors for accurate gene expression in various immune cell types (Mehta and Jeffrey, 2016). Consequently, the immune system of the host is suppressed and this could be one of the ways by which ticks modulate the sophisticated host immune machinery in order to have a successful blood meal (Aounallah et al., 2020). This phenomenon can be exploited by disease causative agents to increase pathogenicity given that epigenetic factors such as DNA or histone modifications are vital for generating a context-specific gene expression in various innate immune cell types (Mehta and Jeffrey, 2015).

In the microscopic image of the rabbit skin site bitten by the TCP dsRNA-injected ticks, increased inflammatory infiltrates/granulocytes (could be predominantly heterophiles/neutrophiles) were observed. This result probably points to the inability of the tick saliva to appropriately modulate or suppress the activities of the host immune molecules when TCP is minimized in or eliminated from the tick saliva. This observation necessitates further investigation to fully unravel the role of TCP during blood acquisition and pathogen transmission.

### Actin Depolymerizing Factor Influences the Speed of Pathogen Motility

Actin depolymerizing factors (ADF) are small actin-binding proteins present in all eukaryotes (Hotulainen et al., 2005). This protein has been previously identified in the saliva of *H. longicornis* (Tirloni et al., 2015) just as in the present study. ADF depolymerize actin filaments at their point ends, thus, facilitating the rate of actin filament turnover and providing a pool of actin monomers for filament assembly (Carlier et al., 1999; Hotulainen et al., 2005). Notably, actin filaments play a key role in cell migration and cytokinesis (Hotulainen et al., 2005). During tick feeding, pathogens could take advantage of the dynamics of ADFs in the tick saliva which provides the pool of actin monomers for filament assembly to fast-track the movement of pathogens from cell to cell (Krause et al., 2003). As microbes gain entrance into the cytoplasm, they recruit host actin and other proteins to their surfaces to induce the assembly of an actin tail; the actin tail provides adequate force to propel them through the cytoplasm of the infected cell and into nearby cells (Gouin et al., 2005). This actin-based motility has been observed in pathogens including spotted fever group *Rickettsia* spp. *Shigella* spp., *Listeria monocytogenes*, and vaccinia virus (Krause et al., 2003; Gouin et al., 2005).

Moreover, after RNA interference of ADF in this study, it was observed that the ticks could not acquire blood after seven days of attachment to the rabbit’s ear unlike the GFP-injected group (control) of ticks. This result points to a possible vital role of ADF in the facilitation of hematophagy in *H. longicornis* ticks. Given that the polymerization and depolymerisation dynamics induced by ADF could provide pushing and pulling forces essential in biological systems, it could be that the knockdown of ADF resulted in the loss of traction required for blood-sucking (Pollard and Cooper, 2009; Gunning et al., 2015). Additionally, the presence of inflammatory infiltrate and moderate haemorrhage were observed at the skin site bitten by ADF dsRNA-injected ticks. These results merit further investigation to fully grasp how they are associated with ADF knockdown.

### Superoxide Dismutase Could Decrease the Oxidative Ability of the Host Phagocytes

Superoxide dismutase (SOD), a vital antioxidant enzyme, was identified as a phosphorylated salivary protein in the present study. SOD belongs to the enzymatic antioxidant system that scavenges superoxide radicals/reactive oxygen species (ROS) (Kwon et al., 2012; Ibrahim et al., 2013). SODs are ubiquitous metalloenzymes that protect aerobic and anaerobic organisms against oxidative stress from superoxide radicals in living cells (Yao et al., 2004; Ibrahim et al., 2013). In this regard, the presence of SOD in the saliva of *H. longicornis* ticks suggest that they play a vital role in the saliva-assisted transmission of pathogens during tick feeding. A previous study suggested that the SOD in the tick saliva neutralizes the oxidative ability of phagocytes at the tick bite site (Bensaoud et al., 2019). Consequently, this creates a conducive atmosphere for pathogen transmission. Notably, phagocytes provide a formidable defense system against invading microbes that pose a threat to the life of the host (Splettstoesser and Schuff-Werner, 2002).

### Programmed Cell Death Protein in Tick Saliva Could Be Associated With Immunoinhibitory Activity

Programmed cell death protein (PD) plays an immunoinhibitory role by negatively regulating T-cell activation through interaction with its ligands PD-L1 and PD-L2 (Sajiki et al., 2021). Interestingly, a putative programmed cell death protein was identified in the saliva of *H. longicornis* in the present study. It is not clear whether this protein originated from the host or the tick, in either case, its presence in the tick saliva underscores the immunoinhibitory activity of tick saliva in the host during blood acquisition. Previous studies have highlighted the immunosuppressive effects of tick saliva in the host which constitutes great physiological significance for tick feeding and pathogen transmission (Mejri et al., 2002; Brossard and Wikel, 2004). The knockdown of PD *via* RNAi in the present study did not produce any significant difference in tick feeding compared to the control group injected with GFP. Also, substantial changes were not observed at the skin site bitten by the PD dsRNA-injected ticks apart from the normal dissociation of the collagen fibers and disruption of the epithelial layer observed in all the experimental groups, which were expected given the disruptive nature of the tick’s hypostome during the skin piercing action at the feeding site. In-depth studies of the functions of PD in tick saliva during tick feeding are required.

### Neutrophiles and Their Contributions in Response to Tick Feeding

Neutrophiles are described as the first line of defence against an infection owing to their ability to phagocytose and kill invading microorganisms, which they destroy by generating reactive oxygen species (ROS) coupled with antimicrobial peptides and proteases (Stuart and Ezekowitz, 2005; Pham, 2008). A substantial concentration of inflammatory infiltrate/granulocytes, predominantly neutrophiles were observed at the tick bite site in the present study. This could have been induced by the early infiltration of T-cells that resulted in the secretion of proinflammatory cytokines in response to tick-bite (Anderson et al., 2017). The corresponding degranulation of invading neutrophiles release enzymes such as serine proteases (Pham, 2008) and myeloperoxidase (MPO) (Zhang et al., 2002), which damage the host tissues and enlarge the bite lesion as a feeding pool, thus facilitating tick blood acquisition (Anderson et al., 2017).

## Conclusion

Phosphoproteomic analysis is vital given the essential role of post-translational modifications in various physiological processes. This study showed the presence of post-translational modification in tick saliva by identifying phosphorylated salivary proteins of *H. longicornis* ticks, underscoring the important roles of tick saliva phosphoproteins during tick feeding and pathogen transmission. The phosphoproteins of *H. longicornis* tick saliva identified in the present study would add to the database catalogue of tick saliva proteins which will enrich future data mining projects in tick saliva research. Additionally, these identified phosphoproteins will add to the data pool of proteins to be screened for possible vaccine candidates, hence contributing significantly to research endeavours for the control of ticks and tick-borne diseases.

## Data Availability Statement

The datasets presented in this study can be found in online repositories. The names of the repository/repositories and accession number(s) can be found in the article/**Supplementary Material**.

## Ethics Statement

The animal study was reviewed and approved by the Animal Ethics Committee of Hebei Normal University (protocol number: 165031).

## Author Contributions

DA wrote the manuscript. NW, DA, and GC conducted the literature, performed the experiments, and prepared all figures and tables. LH, YZ, KW, and ML collected the ticks and analyzed the data. HW and JL designed the experiment, reviewed and edited the manuscript. All authors contributed to the article and approved the submitted version.

## Funding

This project was supported by the National Natural Science Foundation of China (32071510), the Natural Science Foundation of Hebei Province of China (C2021205006), and the Science and Technology Project of Hebei Education Department (ZD2021064).

## Conflict of Interest

The authors declare that the research was conducted in the absence of any commercial or financial relationships that could be construed as a potential conflict of interest.

## Publisher’s Note

All claims expressed in this article are solely those of the authors and do not necessarily represent those of their affiliated organizations, or those of the publisher, the editors and the reviewers. Any product that may be evaluated in this article, or claim that may be made by its manufacturer, is not guaranteed or endorsed by the publisher.

## Supplementary Material

Supplementary Figure S1 Total ions chromatograph (TIC) and base peak chromatograph of proteins identified by mass spectrometry.

Supplementary Figure S2 RNAi silencing validation of target genes in salivary gland **(A)**, ovary **(B)**, and midgut **(C)**. The results are expressed as the means (n = 3) ± SEM.

Supplementary Table S1 Spreadsheet of the total number of phosphorylated tick saliva proteins that were identified.

Supplementary Table S4 Differentially expressed proteins including immune-related proteins of rabbit plasma from the bite site by SPK silenced ticks.
