# Supplementary figures and images for: Phosphoproteomic Analysis of Haemaphysalis longicornis Saliva Reveals the Influential Contributions of Phosphoproteins to Blood-Feeding Success
Source: Front Cell Infect Microbiol. 2022 Jan 18;11:769026. doi: 10.3389/fcimb.2021.769026 (PMC8804221; doi:10.3389/fcimb.2021.769026)

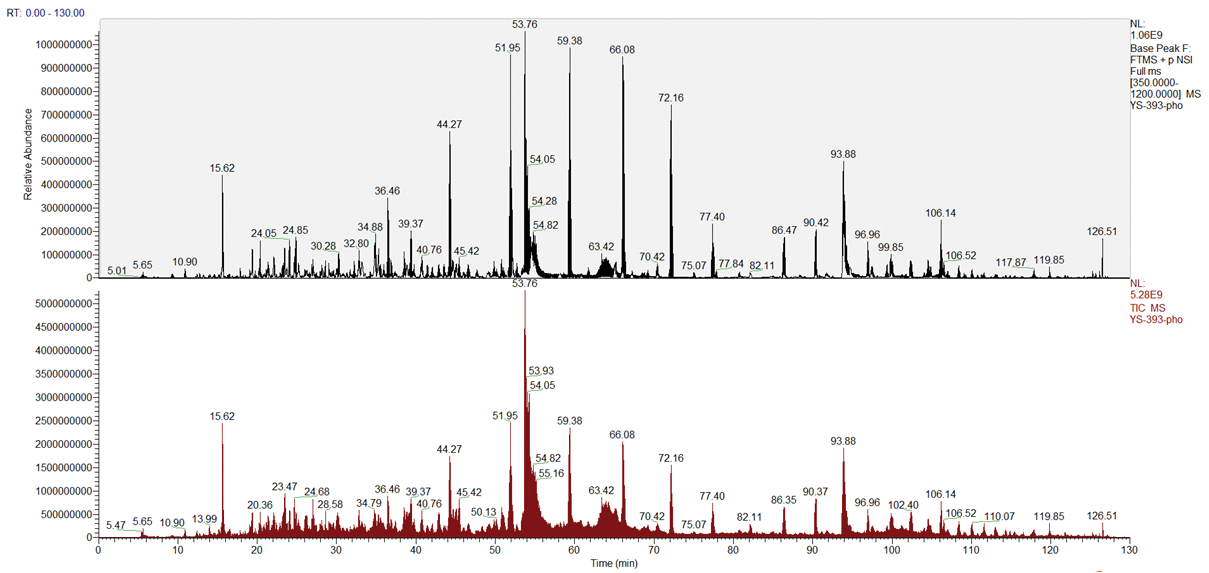

Supplement: Supplementary Figure S1 — Total ions chromatograph (TIC) and base peak chromatograph of proteins identified by mass spectrometry. [file Image_1.tif]

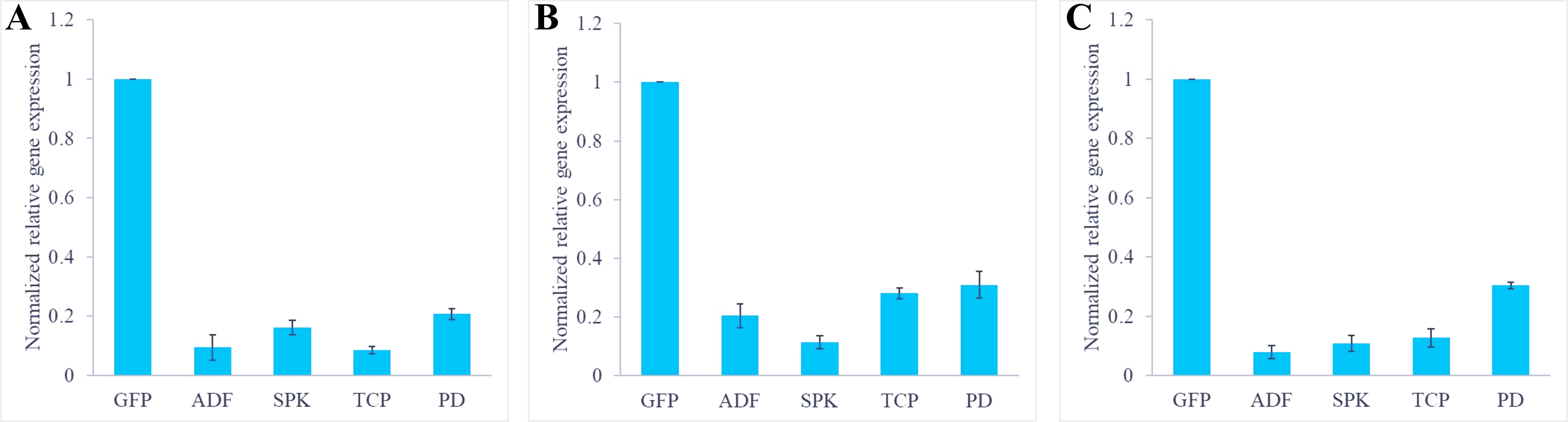

Supplement: Supplementary Figure S2 — RNAi silencing validation of target genes in salivary gland (A), ovary (B), and midgut (C). The results are expressed as the means (n = 3) ± SEM. [file Image_2.tif]
