# Supplementary material for: Phosphoproteomic Analysis of Haemaphysalis longicornis Saliva Reveals the Influential Contributions of Phosphoproteins to Blood-Feeding Success
Source: Front Cell Infect Microbiol. 2022 Jan 18;11:769026. doi: 10.3389/fcimb.2021.769026 (PMC8804221; doi:10.3389/fcimb.2021.769026)
Supplement: Supplementary file 4 [file Table_2.docx]

| **Supplementary Table S2.** Target genes primer sequences used for real-time quantitative PCR | |
| --- | --- |
| **Gene name** | **Primer sequences (5'-3')** |
| *PD* | F: GCTGCGAGCACTGGATG |
|  | R: CGGAACACTTGCCAATAAAC |
| *ADF* | F: CAAGGACGAGCGGGTGA |
|  | R: GGATGGAAGCGGGGAAG |
| *TCP* | F: AGGGACTACGAAATGACGG |
|  | R: GCGGTATCTGGCTCTGG |
| *SPK* | F: AGGTTGACTGCTGGAGTTTAG |
|  | R: TTTCCGCTTTGGCTCATA |
| *ACTIN* | F: CGTTCCTGGGTATGGAATCG |
|  | R: TCCACGTCGCACTTCATGAT |
