# Supplementary material for: Phosphoproteomic Analysis of Haemaphysalis longicornis Saliva Reveals the Influential Contributions of Phosphoproteins to Blood-Feeding Success
Source: Front Cell Infect Microbiol. 2022 Jan 18;11:769026. doi: 10.3389/fcimb.2021.769026 (PMC8804221; doi:10.3389/fcimb.2021.769026)
Supplement: Supplementary file 5 [file Table_3.docx]

| **Supplementary Table S3.** Vitellin primer sequences for real-time quantitative PCR | |
| --- | --- |
| **Gene name** | **Primer sequences (5'-3')** |
| *Vitellin 1* | F: CGTCATCCCCTCAGACAA |
|  | R: ACCTCCCGCTGCTTACCC |
| *Vitellin 2* | F: CGCCAGCTAACGAATGC |
|  | R: CACCAGTAGTTGTAGAAGACGC |
| *ACTIN* | F: CGTTCCTGGGTATGGAATCG |
|  | R: TCCACGTCGCACTTCATGAT |
