# Supplementary material for: Phosphoproteomic Analysis of Haemaphysalis longicornis Saliva Reveals the Influential Contributions of Phosphoproteins to Blood-Feeding Success
Source: Front Cell Infect Microbiol. 2022 Jan 18;11:769026. doi: 10.3389/fcimb.2021.769026 (PMC8804221; doi:10.3389/fcimb.2021.769026)
Supplement: Supplementary file 7 [file Table_5.docx]

| **Supplementary Table S5.** The number of ticks that attached and engorged from the total number of ticks fed on the rabbit host after RNAi of the ticks. | | |
| --- | --- | --- |
| **dsRNA-injected ticks** | **Total Number of ticks fed on the host** | **Number of ticks that engorged** |
| ADF silenced ticks | 25 | 0 |
| SPK silenced ticks | 25 | 20 |
| TCP silenced ticks | 25 | 15 |
| PD silenced ticks | 25 | 22 |
| dsRNA-GFP ticks (control) | 25 | 23 |
